# Preparation of Ag-Decorated TiO_2_ Composite Materials and Study on Photocatalytic Performance

**DOI:** 10.3390/nano15181383

**Published:** 2025-09-09

**Authors:** Hongfei Dou, Jie Wang, Yan Zhao, Junjie Liu, Yannan Li

**Affiliations:** 1 School of Physical Science and Technology, Inner Mongolia University, Hohhot 010021, China; 32346098@mail.imu.edu.cn; 2 College of Energy Materials and Chemistry, Inner Mongolia University, Hohhot 010021, China; 22491023@mail.imu.edu.cn (J.W.); yanzhao@imu.edu.cn (Y.Z.)

**Keywords:** TiO_2_, Ag decorating, photocatalytic antibacterial

## Abstract

Aiming at the insufficient broad-spectrum absorption and high carrier complexation rate in the photocatalytic antimicrobial application of TiO_2_, Ag/TiO_2_ composite materials were prepared by co-precipitation method in this study. The material characterization showed that Ag was uniformly dispersed on the TiO_2_ surface in the form of nanoparticles, and the specific surface area of Ag/TiO_2_ composite materials was enhanced by 59.6% compared with that of pure TiO_2_, and the mesoporous structure was significantly optimized. Visible photocatalytic tests showed that the degradation rate of Ag/TiO_2_ composite materials for Rh B and M O was more than two times higher than that of pure TiO_2_. Under dark conditions, the material showed a minimum inhibitory concentration (MIC) of 62.5 μg/mL against Escherichia coli and Staphylococcus aureus, with an antimicrobial rate of 99.8% for 8 h, confirming its non-light-dependent antimicrobial activity. Mechanistic studies revealed that photogenerated electrons were efficiently captured by Ag nanoparticles, which inhibited e-h^+^ complexation; meanwhile, the photothermal effect (ΔT > 15 °C) promoted the sustained release of Ag^+^, which realized the triple synergistic antimicrobial activity by disrupting the bacterial membrane and interfering with metabolism. This study provides a new strategy for the development of efficient solar-powered water treatment materials.

## 1. Introduction

With the continuous development of science and technology in the society, people have put forward higher requirements for antimicrobial hygiene and safety of wastewater treatment equipment, medical supplies, food packaging, and other daily applications [1,2,3]. Photocatalytic antimicrobial technology has gained widespread attention in the field of disinfection and sterilization for its safety and cost-effectiveness. The inorganic material titanium dioxide (TiO_2_) has been recognized as one of the most promising photocatalytic materials, owing to its non-toxicity, environmental friendliness, and stable physicochemical properties [2,3,4,5]. Under UV excitation, TiO_2_ generates hydroxyl radicals (-OH), which significantly inhibit bacterial proliferation by disrupting cell membranes and biomolecules [6]. However, TiO_2_ suffers from a narrow photoresponse spectrum and a high electron-hole pair recombination rate, which restricts its antimicrobial activity to UV irradiation conditions [7,8,9] and hinders its practical implementation across diverse scenarios [1,2,3,10].

Researchers have found that TiO_2_ photocatalysts with visible light activity can be synthesized through doping TiO_2_ with transition metals [11], non-metals [12], or coupling with graphene [13], among other strategies. Among them, metal doping effectively improves the semiconducting properties of TiO_2_ and reduces structural defects [2,3,10] metals such as Au, Ag, Pt and Pb are commonly used for metal modification due to their high Schottky barrier potentials, which effectively inhibit the recombination of electron-hole pairs and improve the photocatalytic efficiency [3,10,14]. Among these metals, silver (Ag) stands out for its low biological toxicity, broad-spectrum antibacterial activity, long-lasting antibacterial effect and is not easy to develop resistance. Compared to other noble metals (e.g., gold and platinum), Ag offers better cost-effectiveness and lower toxicity. Moreover, Ag has strong antibacterial activity against both Gram-positive and Gram-negative bacteria, such as Staphylococcus aureus and Escherichia coli, which are both common water pathogens [15,16]. Therefore, Ag is the first choice for TiO_2_ modification [1,10,14].

**Scheme 1:**
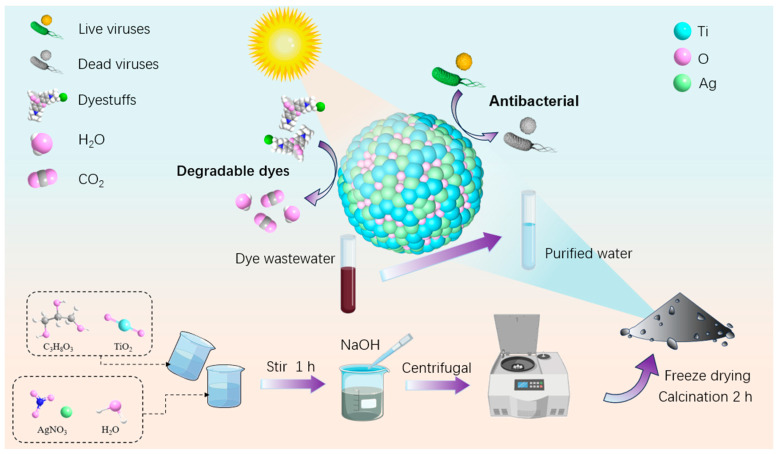
Schematic diagram of the synthesis of Ag/TiO_2_ composite materials.

In this study, Ag/TiO_2_ composite materials were fabricated through an efficient and environmentally friendly co-precipitation method, as shown in Scheme 1. During the preparation process, chemical reagents with low environmental impact were selected and the reaction parameters were optimized to ensure high reaction efficiency while minimizing energy consumption and waste emission. Ag/TiO_2_ composite materials in this paper underwent systematic characterization. The results revealed that their photocatalytic degradation efficiency, antimicrobial properties and photothermal properties were all superior to those of pure TiO_2_. Notably, the excellent photothermal properties of this black composite material can also enhance the original decorated Ag^+^ sterilization under simulated sunlight exposure, achieving synergistic antibacterial effects.

## 2. Experimental Part

### 2.1. Materials

Titanium dioxide (TiO_2_, AR, West Asian reagent), Silver nitrate (AgNO_3_, AR, C = 0.1073 mol/L, Tianjin Xinbote Chemical Co., Ltd., Tianjin, China), Sodium hydroxide (NaOH, AR, Sinopharm Chemical Reagent Co., Ltd., Shanghai, China), Glycerol (C_3_H_8_O_3_, AR, Tianjin Xinbote Chemical Co., Ltd.), Rhodamine B (C_28_H_31_CIN_2_O_3_, Shanghai Aladdin Biochemical Technology Co., Ltd., Shanghai, China), Methyl Orange (C_14_H_14_N_3_N_8_O_3_S, Shanghai Aladdin Biochemical Technology Co., Ltd.), MH Broth (MHB, Beijing Land Bridge Technology Co., Ltd., Beijing, China), LBA gar (LBA gar, Beijing Land Bridge Technology Co., Ltd.), Deionized water (prepared by laboratory pure water meter).

### 2.2. Materials Preparation

In this experiment, 14 mL of glycerol and 10 mL of deionized water were weighed, and then 1 mL, 3 mL and 5 mL of AgNO_3_ standard titration solution were respectively measured into three beaker, TiO_2_ powder was weighed, and the glycerol, AgNO_3_ solution and TiO_2_ powder were integrated into the deionized water to obtain three groups of white turbid liquid with different Ag content. A magnetic agitator was used to stir the TiO_2_ solution containing Ag element evenly, and NaOH solution was dropped into the solution to make the pH value of the solution reach 12. After 1 h of agitation, the solution was centrifuged to obtain brown flocculent precipitation and freeze-dried to obtain fluffy Ag/TiO_2_ composite materials, which was finally sintered in a Muffle furnace at 300 °C for 2 h. Wait for the temperature to cool to 27 °C, grind into powder to prepare Ag/TiO_2_ composite materials, named Ag-1/TiO_2_, Ag-3/TiO_2_ and Ag-5/TiO_2_ respectively (Table S1).

### 2.3. Performance Characterization

In this experiment, a field emission Scanning Electron Microscope (SU8100, Scanning Electron Microscope, SEM), an X-ray diffractometer (Mini Flex 600, Diffraction X-ray rays, Nisaka, Japan, XRD), refinement of XRD was conducted using GSAS 2, version 5782 and a Transmission electron microscope (Model: HT 7800, produced by Hitachi, Japan, TEM) were used. The surface morphology of the materials was observed and the crystal structure of the material was determined. Point scanning of the material was performed with Energy Dispersive Spectrometer (Brucker-QUANTAX 200, EDS, Hitachi Co., Ltd., Tokyo, Japan) to obtain information on the elemental composition. The in-situ X-ray photoelectron spectroscopy system (Thermo Fisher Scientific (China) Co., Ltd., Shanghai, China, ESCALAB XI+) was used to determine the chemical state of elements. The photocatalytic reaction was performed using a photochemical reaction instrument (Shanghai Hefan Instrument Co., Ltd., Shanghai, China, HF-GHX-IV), and the absorbance of the material was determined using an ultraviolet-visible spectrophotometer (PerkinElmer, Shelton, CT, USA, LAMBDA750s) in the appropriate wavelength range. Infrared optical processor (FLUKE, VT04 Visual IR Thermometer) and xenon lamp light source (CEL-HXF300-T3, Beijing China Education Au-Light Technology Co., Ltd., Beijing, China,) were used for optical thermal temperature measurement experiment.

### 2.4. Minimum Inhibitory Concentration Test (MIC)

The antimicrobial effect of Ag/TiO_2_ composite materials was investigated using Gram-negative (Escherichia coli, *E. coli*: ATCC25922), Gram-positive (Staphylococcus aureus, *S. aureus*: ATCC25923) bacteria and (Methicillin-resistance Staphylococcus aureus, *MRSA*:433300). In this experiment, 32 mg of Ag-1/TiO_2_, Ag-3/TiO_2_ and Ag-5/TiO_2_ composite materials and pure TiO_2_ powder were each placed in four centrifuge tubes, and 1 mL of deionized water was added to each centrifuge tube for 15 min. A uniformly dispersed suspension of composites was obtained. Use ultraviolet lamp to irradiate all experimental drugs and utensils for 30 min to sterilize. After the addition of MHB and bacteria, Dilute 32 mg/mL of the composite suspension to 8 mg/mL in a 96-well plate, then dilute each well in half sequentially and incubate it for 12 h in a dark shade. On the next day, the growth of both bacteria in the well plates was observed and counted.

### 2.5. Photocatalytic Degradation Experiment

The photocatalytic activity of all materials was evaluated by degrading Rh B dye solution and M O dye solution under mercury lamp irradiation [17,18,19,20,21]. A 300 W Xenon lamp, mimicking the solar spectrum, was used as the light source. First, 5 mg/L Rh B dye solution and 5 mg/L M O dye solution were prepared. Then, 1 mg of composite materials was weighed and dispersed in the dye solution. The resulting suspensions containing 1 mg composite materials in 45 mL Rh B dye solution and M O dye solution were used for photocatalytic degradation experiments. Prior to testing, the material suspension was stirred in the dark for 30 min to allow the molecules to reach adsorption-desorption equilibrium. The containers were maintained at a fixed distance of 20 cm from the light source throughout the experiment. Two control groups were set up in the experiment. One group involved the photodegradation of Rh B and M O dyes under light without a catalyst, while the other group involved the degradation of the two dye-containing wastewater under dark conditions using a photocatalyst. At intervals of 15 min, the Rh B solution and M O solution were removed from the reaction container for centrifugation, and the supernatant was measured using UV-visible spectrophotometry. The absorption peak intensity was measured and used to plot a light absorption intensity curve to evaluate photocatalytic activity.

### 2.6. Photothermal Imaging Thermal Test

In this experiment, 0.1 g of Ag-1/TiO_2_, Ag-3/TiO_2_ and Ag-5/TiO_2_ composite materials, and pure TiO_2_ powder were each placed in centrifuge tubes. Then, 1 mL of deionized water was added to each tube, and the tubes were ultrasonically dispersed for 5 min to prepare a uniform suspension. Half of the suspension (500 μL) was then placed in each well of a 48-well plate. The suspensions were then irradiated with a 300 W xenon lamp for 65 min, with measurements taken at intervals of 5 min to record temperature changes. Finally, temperature change curves were plotted to evaluate their photothermal performance.

## 3. Results and Discussion

**Figure 1:**
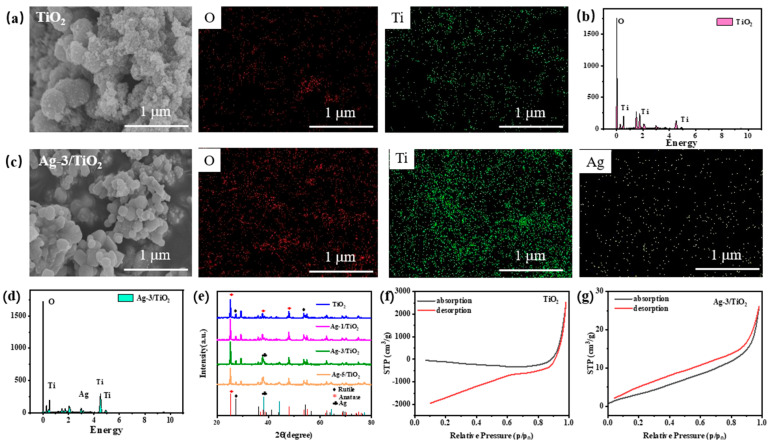
(**a**–**d**) SEM images, mapping images, EDS images, (**e**) XRD patterns, specific surface area curve of (**f**) TiO_2_ and (**g**) Ag/TiO_2_ composite materials.

In order to observe the surface morphology of the materials, we performed SEM tests on TiO_2_ decorated with different concentrations of Ag^+^ (Figure 1a,c). As can be seen from the figures, pure TiO_2_ has obvious agglomeration phenomenon and the grains are piled up together. And with the increase of the concentration of Ag^+^, the aggregation phenomenon of TiO_2_ was obviously reduced, indicating that the doping of Ag^+^ could effectively improve the agglomeration phenomenon of TiO_2_, increase the specific surface area of the composite material, and thereby contribute to enhancing the photocatalytic activity [2,17,18,22]. We believe that by decorating TiO_2_ with Ag, the Ag/TiO_2_ composite material already contains Ag elements. Therefore, to verify our conjecture, we conducted an EDS test on the Ag/TiO_2_ composite material. From the figure (Figure 1a–d), it can be clearly seen that the material contains three elements: O, Ti and Ag, and no other elements, indicating that silver-decorated TiO_2_ has been successfully prepared. Furthermore, TEM and XPS data also confirm the successful modification of TiO_2_ with Ag as shown in Figures S1 and S2.

Similar results were observed in the XRD spectra of the materials (Figure 1e). Compared with the XRD patterns of standard TiO_2_ (PDF anatase #21-1272 and PDF rutile #21-1276), as the concentration of decorated Ag^+^ increased, although the main diffraction peaks of the materials had no shift (diffraction Angle was 25.3°), the characteristic peaks became stronger and stronger. It is well known that the diffraction peak at 25.3° is a characteristic of the TiO_2_ anatase structure. In addition, as shown in Figure 1e, compared with the XRD pattern of standard Ag (PDF#87-07), the diffraction peak of Ag (at a diffraction angle of 38.1°) becomes more distinct due to the increase in Ag^+^ concentration, indicating that Ag has successfully modified TiO_2_. Figure S3 also proves this point.

Compared with pure TiO_2_, the hysteresis line of the specific surface area test of the Ag/TiO_2_ composite materials is more obvious, and the pressure range that appears is also wider, indicating that the materials have a more developed mesoporous structure, which is conducive to increasing the reaction sites of surface reactions (Figure 1f,g). Furthermore, the adsorption curves of the Ag/TiO_2_ composite materials show that the adsorption capacity significantly increases in the low-pressure region, indicating an increase in the microporosity and specific surface area of the materials. Specific values can be found in Table S2. This result is consistent with the aforementioned one. With the increase of Ag ions, the dispersion of the material increases, which is conducive to improving the photocatalytic degradation efficiency of dye wastewater.

**Figure 2:**
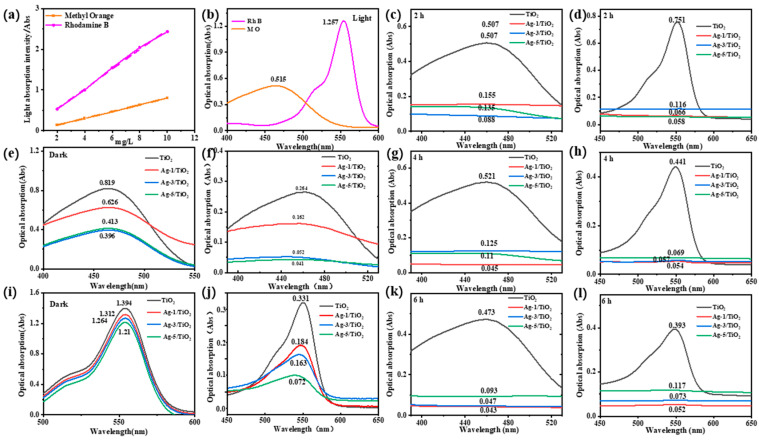
(**a**) Standard curves for Rh B and M O, the degradation curves of M O (**e**) and Rh B (**i**) by the Ag/TiO_2_ composite material within 15 min in the dark, (**b**,**f**,**j**) respectively represent the curves of simulated solar light degradation of dyes, Ag/TiO_2_ composite material degradation of M O and Rh B within 15 min under illumination, light absorption curves for photocatalytic degradation of (**c**,**g**,**k**) Rh B and (**d**,**h**,**l**) M O at 2 h, 4 h and 6 h for Ag/TiO_2_ composite materials.

Then, we evaluated the photocatalytic efficiency of all materials by degrading Rh B and M O dyes. Linear fitting revealed a strong linear correlation between dye mass concentration and absorbance within the range of 0–12 mg/L, which enabled the determination of the photocatalytic activity of Ag/TiO_2_ composite materials (Figure 2a). Please refer to Figure S4 for the spectral irradiance map at the sample location, The dark adsorption effect of the Ag/TiO_2_ composite materials on the dyes is shown in Figure S5.

The absorbance curves of the Ag/TiO_2_ composite materials for the degradation of Rh B and M O dyes (Figure 2f,g) showed that these materials exhibited significantly higher degradation rates than the control group (pure TiO_2_). Notably, the degradation efficiency of TiO_2_ increased progressively with the rise in Ag^+^ concentration.

Compared with the control group (b), by modifying TiO_2_, the Ag/TiO_2_ composite materials exhibited excellent photocatalytic degradation performance for Rh B and M O dyes. The degradation rate data of all materials for Rh B and MO dyes are shown in Table 1. Among all decorated materials, the degradation rates of Ag-3/TiO_2_ on Rh B and M O dye were more than 78%. The decorated Ag^+^ effectively alleviated the aggregation of pure TiO_2_, increased the contact area between Ag/TiO_2_ composite materials and dye molecules, and improved the photocatalytic efficiency [18,23,24]. The above experimental results are also consistent with the statistical results of the specific surface area of the materials (Figure 1f,g and Table S2).

Additionally, the time dependence of dye wastewater degradation for all materials was also investigated through testing. With the increase of the treatment time of dye wastewater to the 2nd, 4th and 8th h, the absorbance of the dye wastewater treated by Ag/TiO_2_ composite materials continued to decrease (Figure 2c,d,g,h,k,l). It also proves that Ag^+^ doping increases the contact area of TiO_2_ with dye molecules. In addition, Ag^+^ doping can reduce the forbidden band width between valence and conduction bands (As shown in Figure S6), which can broaden the photocatalytic response range of TiO_2_, enhance the utilization of visible light, generate strong oxidizing radicals, and, at the same time, inhibit the complexation of photogenerated electrons and holes, thus improving the photocatalytic efficiency [25,26]. Furthermore, Figure S7 shows that the absorption intensity of Ag/TiO_2_ is significantly higher than that of TiO_2_, which also confirms that the addition of Ag to TiO_2_ enhances the utilization of visible light and thereby improves the photocatalytic efficiency.

As widely acknowledged in literature, ref. [27] the presence of Ag^+^ contributes to the enhancement of the material’s antibacterial properties. In this study, the antibacterial activities of the prepared materials against *E. coli* and *S. aureus* were evaluated by using the bacterial suspension in the wells as the control group and other materials as the experimental group. Pure TiO_2_ exhibited no antibacterial activity under dark conditions. By comparison, Ag/TiO_2_ composite materials demonstrated superior antimicrobial performance in the dark. The minimum inhibitory concentration (MIC) decreased with the increase of Ag^+^ content, indicating an enhanced antibacterial ability (Table 2). The experimental results confirmed that the Ag/TiO_2_ composite materials significantly improved the antibacterial effect against Escherichia coli and Staphylococcus aureus under dark conditions as shown in Figure S8.

**Figure 3:**
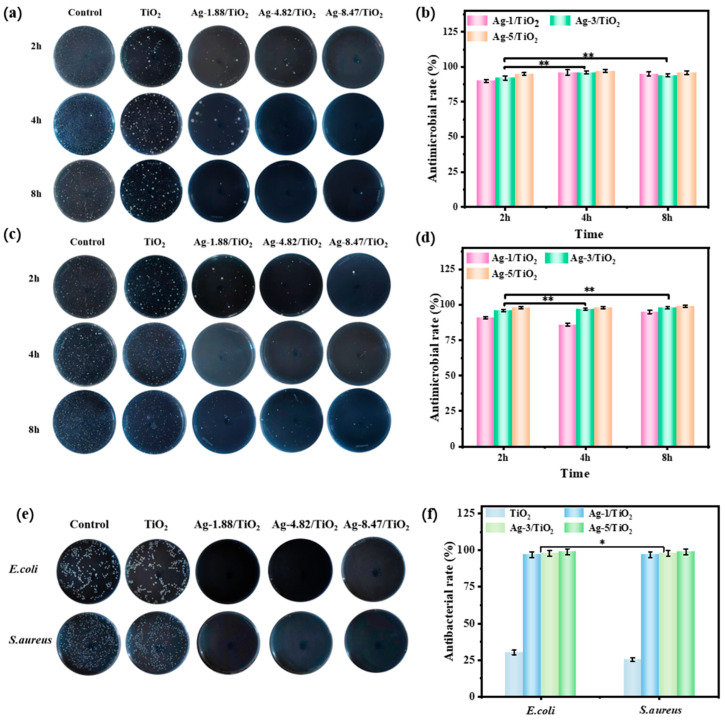
(**a**) Plate count graphs of *E. coli* and (**c**) *S. aureus* under dark conditions, (**b**,**d**) the corresponding statistical graphs of antibacterial rates of Ag/TiO_2_ composite materials, (**e**) plate count graphs of *E. coli* and *S. aureus* under light conditions, (**f**) the corresponding statistical graphs of antibacterial rates of Ag/TiO_2_ composite materials. All statistical data are represented as mean ± SD; *n* = 3. The significance of differences was treated as follows: * represents *p* < 0.05, ** represents *p* < 0.01.

Among all the experimental materials, Ag-5/TiO_2_ showed the best antimicrobial property against *E. coli* and *S. aureus* with a minimum inhibitory concentration of 62.5 μg/mL under dark conditions, while pure TiO_2_ powder had no antimicrobial effect as shown in Figure S8. The superior antimicrobial property of Ag/TiO_2_ composite materials was attributed to the presence of Ag^+^, which as the Ag^+^ concentration increased, the probability of electron trapping enhanced, inhibiting electron-hole pair recombination, disrupting microbial structure, and interfering with bacterial metabolic processes—thereby conferring robust physical antimicrobial properties [28]. The TEM data of bacteria and samples also confirm this, as shown in Figure S9. In addition, the antibacterial effect released by the material in the solution was tested (Figure 3a–d). As can be seen from the colony growth status control plots (Figure 3a,c): the number of colonies in the Control group (pure bacteria) and the TiO_2_ group increased significantly at the 2 h, 4 h, and 8 h time points, indicating that the antimicrobial performance of the silver-free materials was limited; while the colony density of Ag/TiO_2_ composites materials (Ag-1/TiO_2_, Ag-3/TiO_2_, and Ag-5/TiO_2_) decreased in a gradient with the increase of Ag loading. Ag-5/TiO_2_ showed a gradient decrease in colony density with increasing Ag loading, and the Ag-5/TiO_2_ group had almost no visible colonies at 8 h. The results showed a gradient decrease in colony density with increasing Ag content. Quantitative antimicrobial rate analysis (Figure 3b,d) showed that the antimicrobial rates of all silver-containing materials were greater than 90% within 2 h and increased with time. Among them, the antimicrobial rate of Ag-5/TiO_2_ was as high as 99.8% at 8 h, which was significantly better than that of the low-silver loading group (Ag-1/TiO_2_: 93.2%, Ag-3/TiO_2_: 97.5%), confirming that the antimicrobial activity increased with the increase of Ag concentration. In addition, we also conducted MIC tests on the drug-resistant bacterium MRSA and carried out statistical analysis on it as shown in Figure S10. The experimental results prove that when Ag/TiO_2_ is faced with more complex bacterial species, it can also achieve relatively good antibacterial effects. Please refer to Table S3 for details.

**Figure 4:**
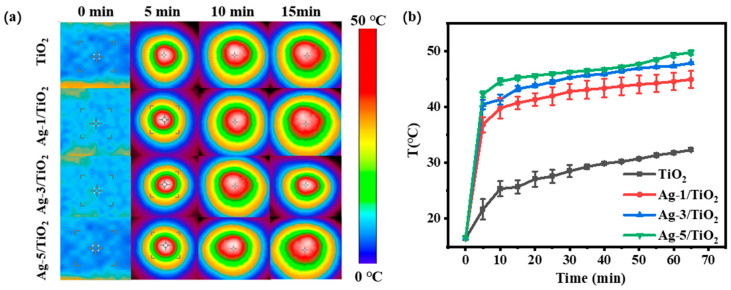
(**a**) Ag/TiO_2_ composite materials photothermal temperature measurement curve, (**b**) Ag/TiO_2_ composite materials photothermal imaging temperature rise map.

As shown in Figure 4a,b, the photothermal performance of Ag/TiO_2_ composite materials exhibits a dual dependence on time and concentration: under simulated sunlight, the temperature of the suspension continues to rise with increasing irradiation time, and the temperature increase (ΔT) is positively correlated with the Ag concentration. The photothermal response of Ag-5/TiO_2_ is the most significant, with the solution center temperature reaching 45 °C (ΔT > 15 °C) after 30 min of irradiation, while pure TiO_2_ only increased by 15 °C. This localized thermal effect enhances antimicrobial performance through a dual pathway: Accelerated dynamic release of Ag^+^: Increased temperature significantly enhances the ion release kinetics of Ag nanoparticles (Arrhenius effect), resulting in a more than 40% increase in the concentration of Ag^+^ released per unit time [29]; Disrupting bacterial metabolic homeostasis: The 45 °C environment disrupts the expression of heat shock proteins (HSPs) in microorganisms, leading to the inactivation of key enzymes and increased cell membrane fluidity [30], which synergizes with the membrane-perforating effect of Ag^+^ to cause rapid bacterial death. Consistent with literature reports [26,31,32], the local surface plasmon resonance (LSPR) effect of metallic Ag is the primary cause of photothermal conversion; in addition, we also demonstrated that photothermal effect can promote the release of Ag^+^ through the Ag^+^ concentration release test as shown in Figure S11b. the synergistic interaction between photothermal and photocatalytic processes (e.g., heat-enhanced carrier mobility) further enhances pollutant degradation efficiency. Furthermore, for environmental protection considerations, we also tested the recyclability of Ag/TiO_2_. The test results show (Figures S11b and S12) that after 5 cycles, the recovery rate of Ag/TiO_2_ is above 95%, which proves that Ag/TiO_2_ has cyclic stability and reusability.

## 4. Conclusions

In conclusion, this study successfully established an Ag/TiO_2_ composite materials photocatalytic antibacterial system. The introduction of Ag enhances performance through two pathways: (1) Structural optimization: Ag nanoparticles inhibit TiO_2_ agglomeration, increasing specific surface area and reaction sites, thereby accelerating pollutant adsorption and degradation; (2) Functional synergy: Photocatalysis: Ag acts as an electron trap to promote e^−^-h^+^ separation, enhancing ROS generation efficiency; sustained antibacterial activity: Ag^+^ release disrupts bacterial membrane structure (dark state MIC: 62.5 μg/mL); photothermal assistance: localized heating (45 °C) accelerates Ag^+^ release, achieving an 8-h antibacterial rate of 99.8%. Experiments show that the Ag-5/TiO_2_ material exhibits optimal performance, with dye degradation rates exceeding 90% and highly efficient broad-spectrum antibacterial activity. This material overcomes the limitations of traditional TiO_2_ narrow spectral response and low quantum efficiency through a synergistic mechanism of photocatalysis-ion release-photothermal heating, providing a feasible solution for solar-driven treatment of dye wastewater containing bacteria.

## Figures and Tables

**Table 1:** Dye degradation rate of Ag/TiO_2_ composite materials (*n* = 3).

Time	Sample Name	Rh B Degradation Rate (%)	M O Degradation Rate (%)
2 h	TiO_2_	60.27	-
	Ag-1/TiO_2_	87.85	59.32
	Ag-3/TiO_2_	93.10	76.90
	Ag-5/TiO_2_	89.42	65.57
4 h	TiO_2_	61.17	-
	Ag-1/TiO_2_	90.20	86.81
	Ag-3/TiO_2_	96.47	88.19
	Ag-5/TiO_2_	91.38	81.13
6 h	TiO_2_	62.93	-
	Ag-1/TiO_2_	96.31	87.66
	Ag-3/TiO_2_	96.63	88.71
	Ag-5/TiO_2_	92.71	85.59

**Table 2:** Statistics of minimum inhibitory concentration of Ag/TiO_2_ composite materials (*n* = 3).

Culture	Sample Name	MIC (μg/mL)
*E. coli*	TiO_2_	0
	Ag-1/TiO_2_	250
	Ag-3/TiO_2_	125
	Ag-5/TiO_2_	62.5
*S. aureus*	TiO_2_	0
	Ag-1/TiO_2_	500
	Ag-3/TiO_2_	125
	Ag-5/TiO_2_	62.5

## Supplementary Materials

The following supporting information can be downloaded at: https://www.mdpi.com/article/10.3390/nano15181383/s1, Figure S1: (a) TEM of Ag/TiO_2_ composite material, the scale bar in the figure represents 200 nm. (b) The corresponding particle size distribution chart.; Figure S2: XPS of (a) TiO_2_ and Ag/TiO_2_, (b) O 1s, (c) Ti 2p and (d) Ag 3d.; Figure S3: The XRD pattern after fine-tuning, (a) TiO_2_, (b) Ag/ TiO_2_.; Figure S4: Spectral irradiance and irradiance at the samples.; Figure S5: Dark adsorption of (a) Rh B and (b) M O by Ag/TiO_2_ composite materials.; Figure S6: The band gap width of TiO_2_ and Ag/TiO_2_.; Figure S7: The UV-Vis spectrum of TiO_2_ and Ag/TiO_2_.; Figure S8: The MIC values against (a) *E.coli* and (b) *S.aureus* statistical analysis.; Figure S9: TEM of bacteria after treatment (membrane damage). The scale bar in the figure represents 1 μm.; Figure S10: The MIC values against *MRSA* statistical analysis.; Figure S11: The concentration of Ag^+^ in solution, (a) The precipitate form Ag^+^ and NaOH, (b) The concentration of Ag^+^ released before and after light exposure.; Figure S12: Reusability of Ag/TiO_2_ composite materials.; Table S1: The weight percent of silver in the samples.; Table S2: BET surface area values of the samples.; Table S3: Statistics of minimum inhibitory concentration of Ag/TiO_2_ composite materials. (*n* = 3).
