# Supplementary material for: Preparation of Ag-Decorated TiO2 Composite Materials and Study on Photocatalytic Performance
Source: Nanomaterials (Basel). 2025 Sep 9;15(18):1383. doi: 10.3390/nano15181383 (PMC12472790; doi:10.3390/nano15181383)
Supplement: Supplementary file 1 [file nanomaterials-15-01383-s001.zip › nanomaterials-3845025-supplementary.pdf]

Preparation of Ag-Decorated TiO_2 Composite Materials and Study on Photocatalytic Performance

Supporting information

Hongfei Dou[a], Jie Wang[b], Yan Zhao[b], Jun-Jie Liu[a*], Yannan Li[a*]

[a] School of Physical Science and Technology, Inner Mongolia University, Hohhot 010021, P.R. China

[b] College of Energy Materials and Chemistry, Inner Mongolia University, Hohhot 010021, P.R. China

[*] Corresponding author.

E-mail address: liyannan@imu.edu.cn (Y.N. Li); pyljj@imu.edu.cn (Jun-Jie Liu).

2.7 Specific surface area test

Take an appropriate amount of sample and place it in a bulb tube. Put the bulb tube into the specific surface area tester. Degas it in a nitrogen atmosphere at 300℃ for 6 h, and then start the specific surface area test. The specific surface area model is BJH.

2.8 Ag^+ concentration release test

A certain mass of Ag/TiO_2 composite material was dispersed in deionized water. Then, the composite material water solution was stirred under dark and light conditions respectively. At the time points of 5 min, 20 min, 40 min and 60 min, 5 ml of the composite material suspension was taken out for centrifugation, and the supernatant was collected. Then, an equal volume of NaOH solution with a pH value of 12 was added to the supernatant. Subsequently, the supernatant containing NaOH was centrifuged, the precipitate was observed and photographed for record. Finally, the precipitate was collected and dried, and the weight of the precipitate was measured. The mass of silver in the precipitate was calculated based on the weight of the precipitate to obtain the concentration of Ag^+ in the solution.

2.9 Bacterial transmission electron microscopy test

After co-culturing the bacteria with the material, 1 ml of the bacterial-sample mixture was centrifuged, the supernatant was discarded, and the precipitate was washed three times with PBS. The precipitate was then fixed overnight with 2.5% glutaraldehyde, followed by centrifugation to discard the fixative and washing the precipitate three times with PBS. The precipitate was dehydrated stepwise with ethanol and finally dispersed in anhydrous ethanol solution for testing.

2.10 *Reusable test*

A certain amount of the composite material was dispersed in the dye solution and stirred thoroughly under xenon lamp irradiation. Centrifugation was performed on the solution every 15 min, the supernatant was discarded, and the precipitate was retained.

After drying the precipitate, its weight was measured. The mass after 5 cycles was statistically analyzed.

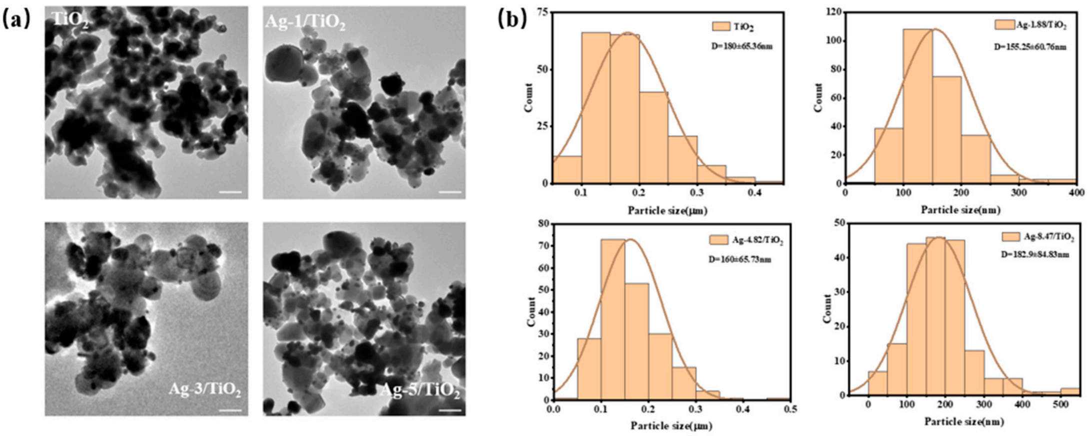

Figure S1. (a) TEM of Ag/TiO2 composite material, the scale bar in the figure represents 200 nm. (b) The corresponding particle size distribution chart.

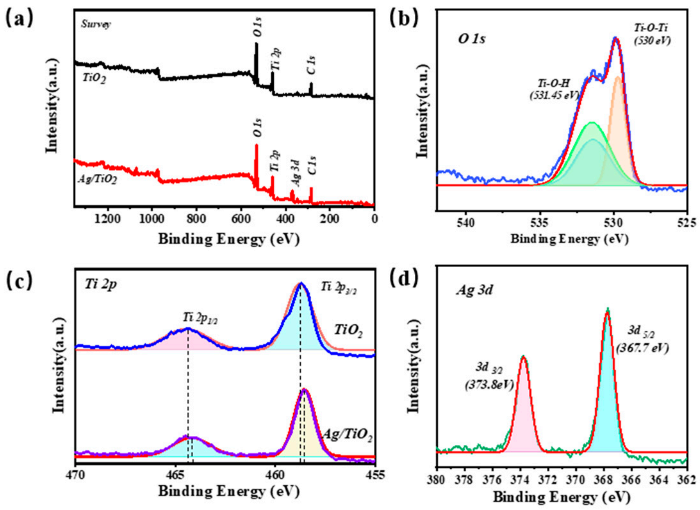

Figure S2. XPS of (a) TiO2 and Ag/TiO2, (b) O 1s, (c) Ti 2p and (d) Ag 3d.

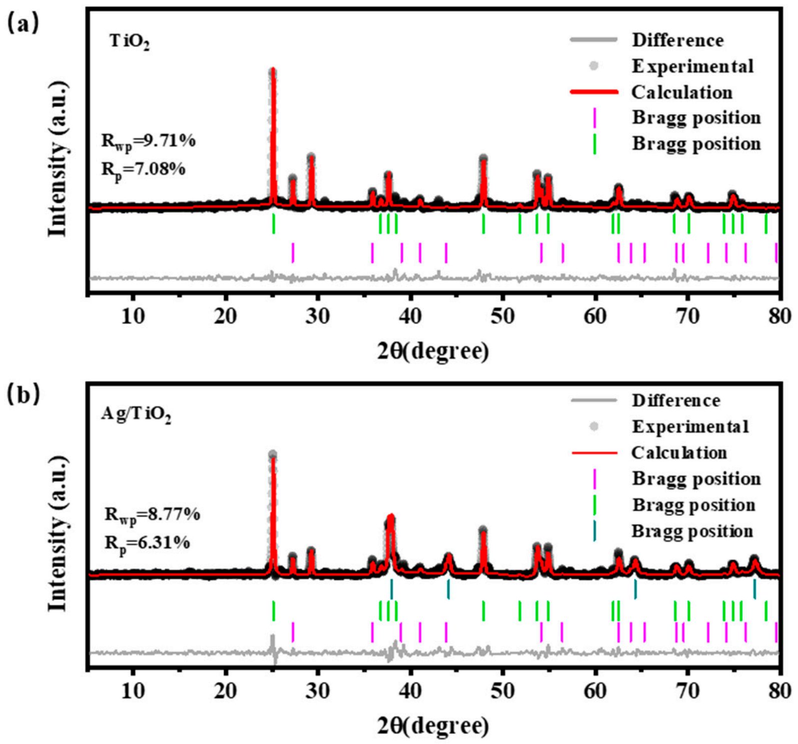

Figure S3. The XRD pattern after fine-tuning, (a) TiO_2, (b) Ag/ TiO_2.

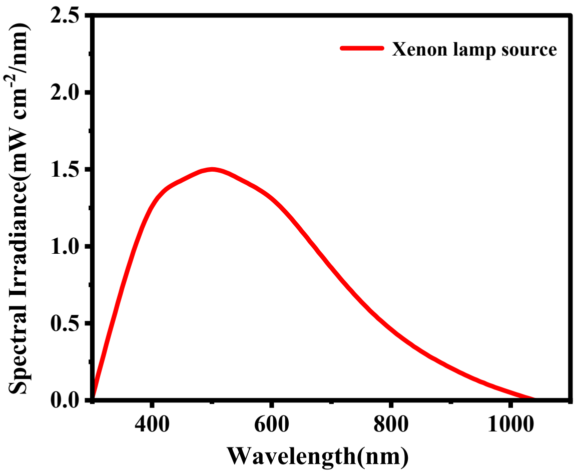

Figure S4. Spectral irradiance and irradiance at the samples.

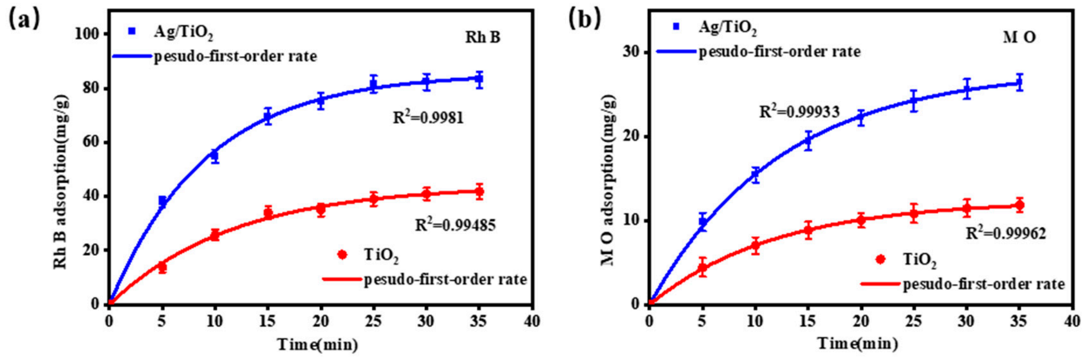

Figure S5. Dark adsorption of (a) Rh B and (b) M O by Ag/TiO_2 composite materials.

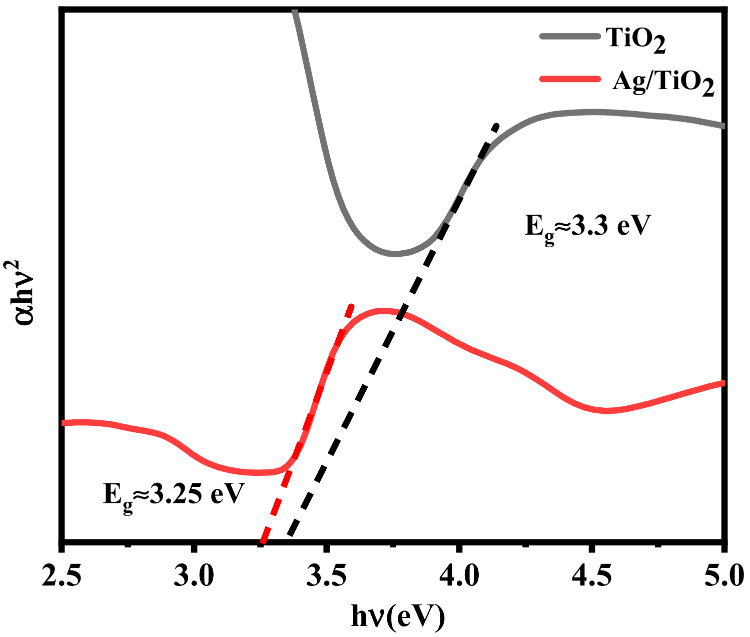

Figure S6. The band gap width of TiO_2 and Ag/TiO_2.

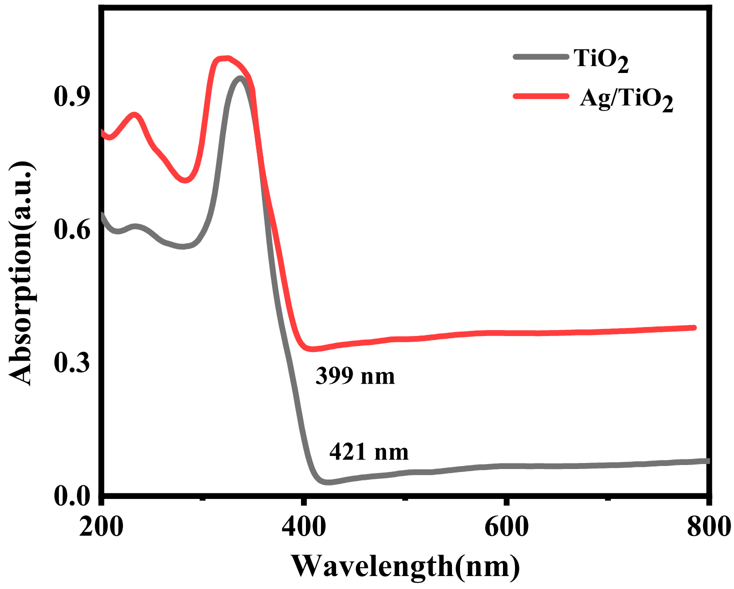

Figure S7. The UV-Vis spectrum of TiO_2 and Ag/TiO_2.

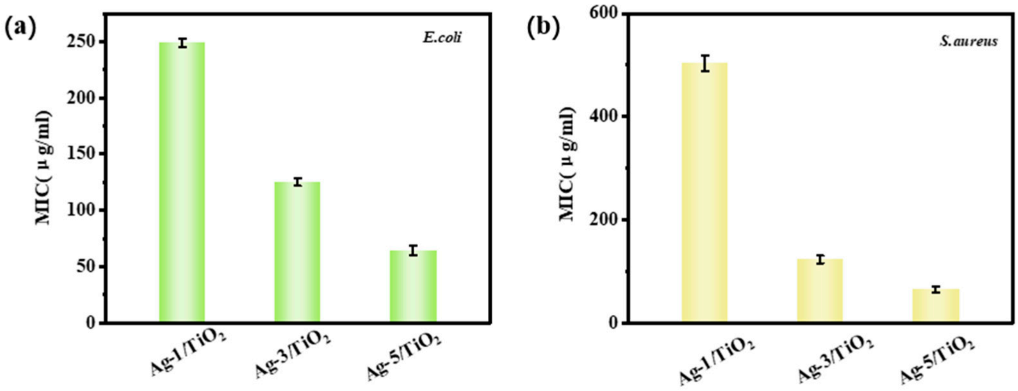

Figure S8. The MIC values against (a) *E.coli* and (b) *S.aureus* statistical analysis.

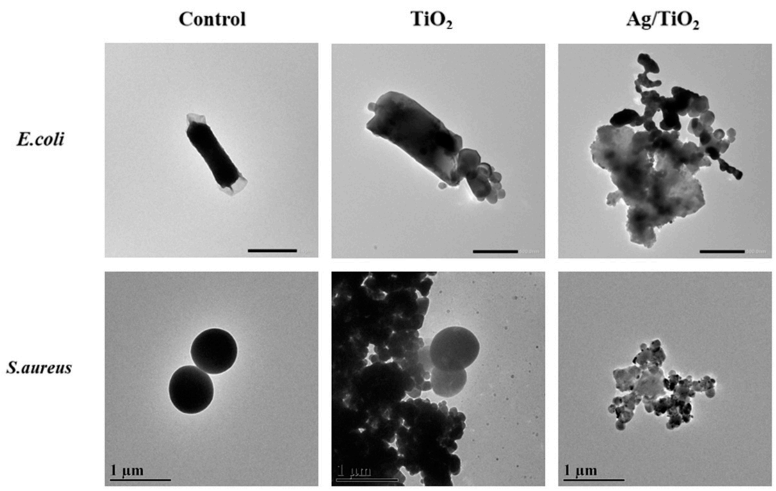

Figure S9. TEM of bacteria after treatment (membrane damage). The scale bar in the figure represents 1 μm.

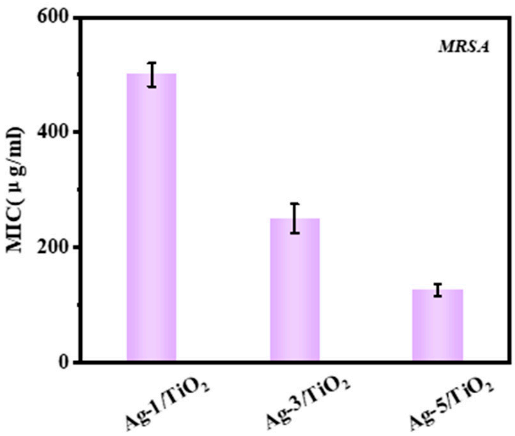

Figure S10. The MIC values against *MRSA* statistical analysis.

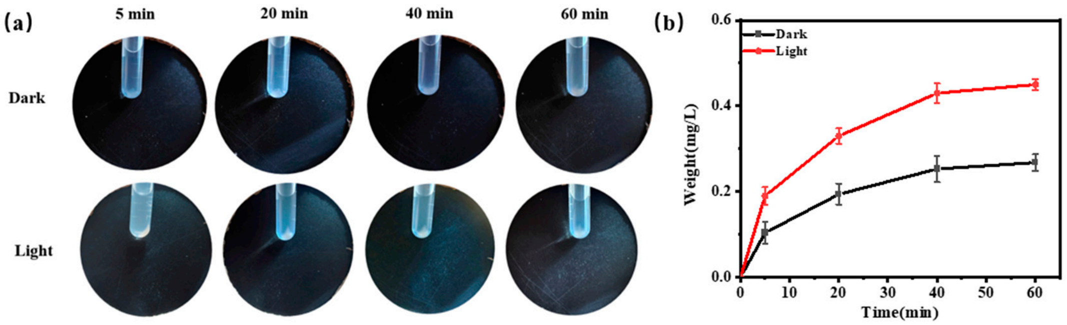

Figure S11. The concentration of Ag^+ in solution, (a) The precipitate form Ag^+ and NaOH, (b) The concentration of Ag^+ released before and after light exposure.

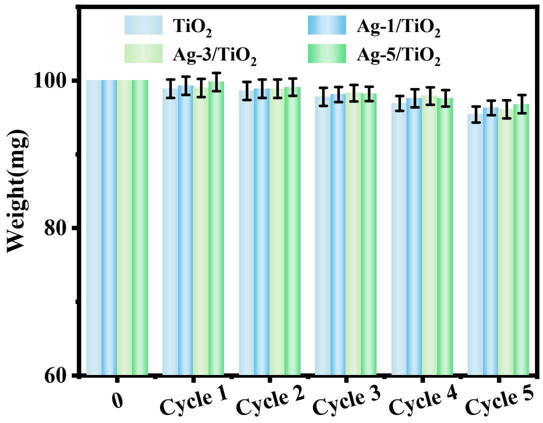

Figure S12. Reusability of Ag/TiO_2 composite materials.

Table 1. The weight percent of silver in the samples.

Sample name	Silver content (wt%)
TiO_2	0
Ag-1/TiO_2	1.88
Ag-3/TiO_2	4.82
Ag-5/TiO_2	8.47

Table S2. BET surface area values of the samples.

Sample name	S_BET	V_pore (cm^{-3}/g)	D_pore (nm)	Isotherms	adsorption-desorption hysteresis
TiO_2	0	4.819	26.789	-	-
Ag-1/TiO_2	11	0.159	11.359	IV	H3
Ag-3/TiO_2	16	0.038	3.412	IV	H3
Ag-5/TiO_2	18	0.032	3.063	IV	H3

Table S3. Statistics of minimum inhibitory concentration of Ag/TiO_2 composite materials. (n = 3)

Culture	Sample name	MIC (mg/ml)
MRSA	TiO_2	0
	Ag-1/TiO_2	0.5
	Ag-3/TiO_2	0.25
	Ag-5/TiO_2	0.125
